# Supplementary material for: Recent African strains of Zika virus display higher transmissibility and fetal pathogenicity than Asian strains
Source: Nat Commun. 2021 Feb 10;12:916. doi: 10.1038/s41467-021-21199-z (PMC7876148; doi:10.1038/s41467-021-21199-z)
Supplement: Supplementary file 1 — Supplementary Information [file 41467_2021_21199_MOESM1_ESM.pdf]

Supporting information

Supplementary File 1. Custom code used to perform epidemiological simulations. The file contains the R script and complete instructions for installing and running the script.

Supplementary Table 1. ZIKV strains included in this study. The name of the ZIKV strains used in the study with their country of origin, original strain name, source of isolation, year of collection and the passage history prior to use in this study are indicated. TS: *Toxorhynchites splendens* mosquitoes; C6/36: *Aedes albopictus* cells; Vero: green monkey kidney cells; SM: suckling mouse brains.

Name in this study	Country	Strain ID	Source	Year	Passage history
F_Polynesia_2013	French Polynesia	PF13/251013-18	Human serum	2013	C6/36-4
Puerto_Rico_2015	Puerto Rico	PRVABC59	Human serum	2015	Vero-2; C6/36-3
Philippines_2012	Philippines	CPC-0740	Human serum	2012	TS-1; C6/36-2; Vero-1; C6/36-1
Thailand_2014	Thailand	SV0127-14	Human serum	2014	TS-1; C6/36-2; Vero-1; C6/36-1
Cambodia_2010	Cambodia	FSS13025	Human serum	2010	Vero 2; SM-1; C6/36-1
Senegal_2011	Senegal	Kedougou2011	Pool of *Aedes* spp. and *Mansonia* spp.	2011	C6/36-2
Senegal_2015	Senegal	Kedougou2015	Pool of *Aedes* spp. and *Mansonia* spp.	2015	C6/36-2

Supplementary Table 2. Parameter estimates of mosquito-to-human transmission events. The transmission kinetics of each ZIKV strain were modeled with a two-parameter log-logistic function to estimate their extrinsic incubation period (EIP) distribution. In the epidemiological simulations, the probability of mosquito-to-human transmission for each contact between a human and an infected mosquito was determined by the transmission probability inferred for each ZIKV strain from the EIP log-logistic distributions of location = log(*e*), and scale = 1/*b*.

	**Log-logistic parameter estimates**	
**ZIKV strain**	*e*	*b*
F_Polynesia_2013	16.60	–10.83
Puerto_Rico_2015	20.74	–8.17
Thailand_2014	18.76	–24.86
Philippines_2012	19.01	–5.72
Cambodia_2010	17.66	–4.99
Senegal_2015	12.05	–3.24

Supplementary Table 3. Oligonucleotides used in this study.

Application	Oligo	Sequence (5'-3')
ZIKV RNA quantification in mosquito experiments	Forward Primer	GTATGGAATGGAGATAAGGCCCA
	Reverse primer	ACCAGCACTGCCATTGATGTGC
ZIKV RNA quantification in mouse experiments (Asian strains)	Forward primer	CCGCTGCCCAACACAAG
	Reverse primer	CCACTAACGTTCTTTTGCAGACAT
	Probe	6FAM/AGCCTACCT/ZEN/TGACAAGCAATCAGACACTCAA/3'IABkFQ
ZIKV RNA quantification in mouse experiments (African strains)	Forward primer	GTCGCTGTCCAACACAAG
	Reverse primer	CACCAGTGTTCTCTTGCAGACAT
	Probe	6FAM/AGCCTACCT/ZEN/TGACAAGCAATCAGACACTCAA/3'IABkFQ
ZIKV RNA standards in mouse experiments (Asian strains)	gBlock	GAGGCATCAATATCAGACATGGCTTCTGACAGCCGCTGCCCAACACAAGGTGAAGCCTACCTTGACAAGCAATCAGACACTCAATATGTCTGCAAAAGAACGTTAGTGGACAGAGGCTGGGGAAATGGATGTGGACT
ZIKV RNA standards in mouse experiments (African strains)	gBlock	GAGGCATCAATATCGGACATGGCTTCGGACAGTCGCTGTCCAACACAAGGTGAAGCCTACCTTGACAAGCAATCAGACACTCAATATGTCTGCAAGAGAACACTGGTGGATAGAGGTTGGGGAAATGGGTGTGGACT

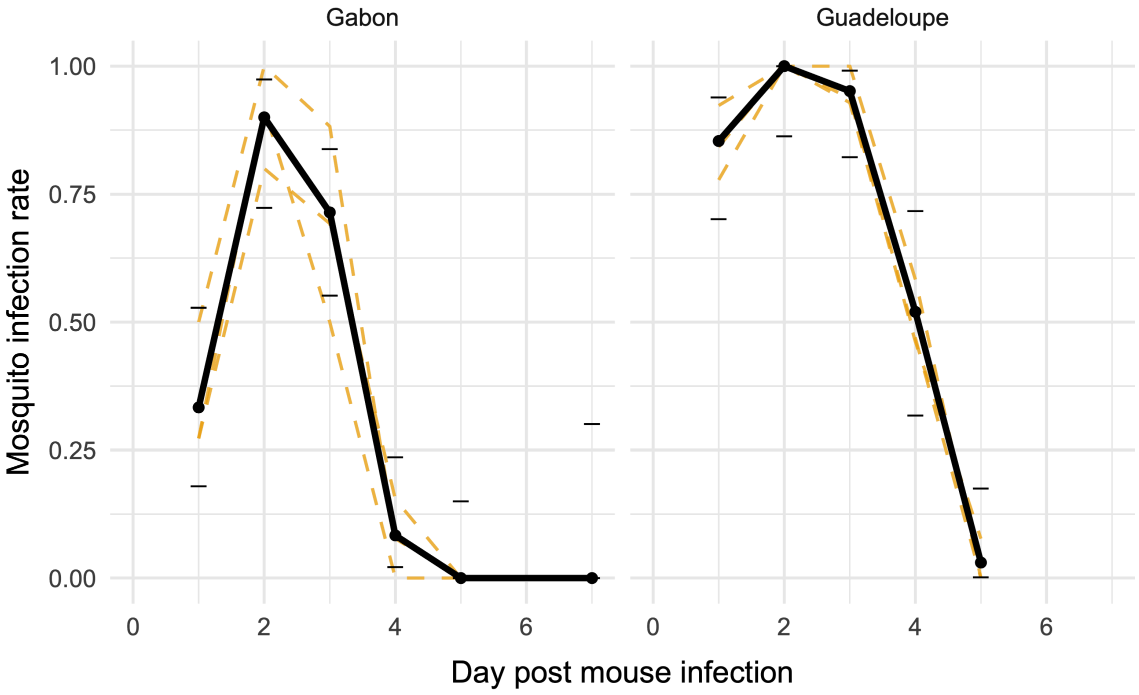

Supplementary Fig. 1. Mouse-to-mosquito ZIKV transmission probability over time. The lines represent the proportion of *Ae. aegypti* mosquitoes from Gabon (left) or Guadeloupe (right) that tested ZIKV-positive 14 days after taking a blood meal on the same triplet of *Ifnar1*$^{-/-}$ mice inoculated with 10^5 FFU of the Cambodia_2010 ZIKV strain on day 0. A median of 11 females per time point and per mouse (range 2-17) were tested for the Gabon population. A median of 13 females per time point and per mouse (range 0-19) were tested for the Guadeloupe population. The gold dashed lines represent the transmission probabilities for the 3 individual mice during their viremic period and the black line represent their mean. Horizontal ticks indicate the 95% confidence intervals of the probabilities. Source data are provided as a Source Data file.

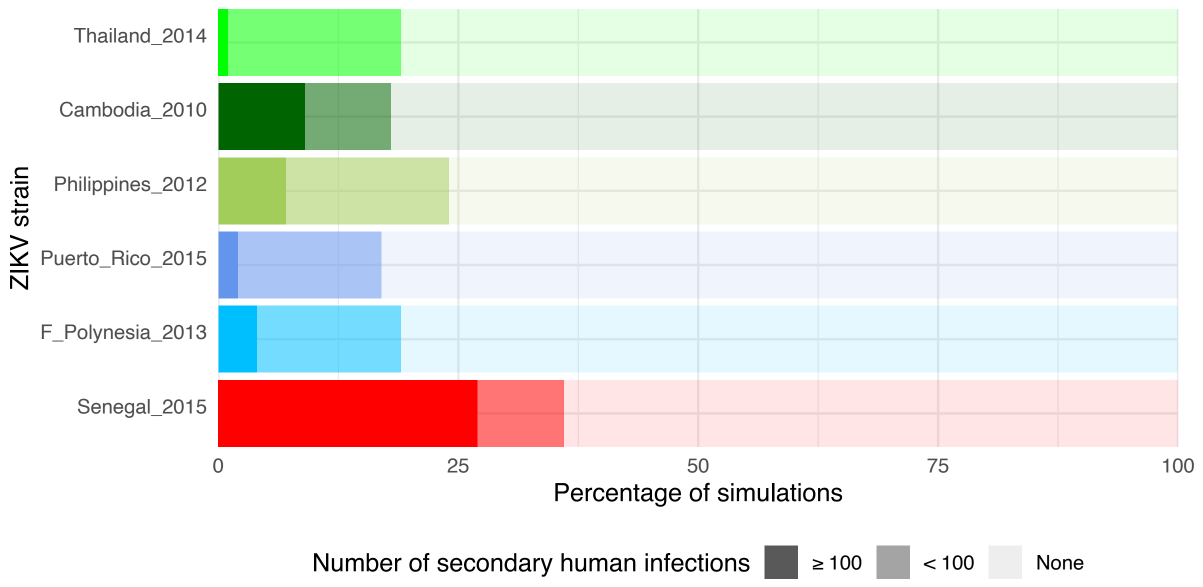

Supplementary Fig. 2. Simulated effect of empirical variation in ZIKV transmissibility on the risk and magnitude of human outbreaks with reduced mosquito susceptibility. The figure shows the simulations results of Fig. 3 when the highly ZIKV susceptible mosquito population from Guadeloupe is replaced with a less susceptible mosquito population from Gabon (Fig. S1) to parameterize human-to-mosquito transmission events.

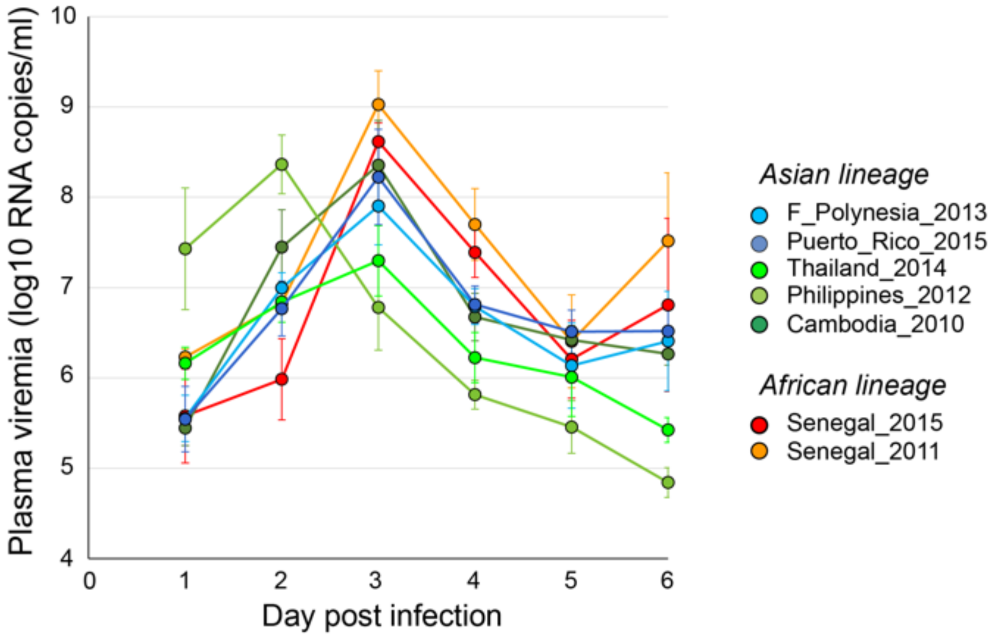

Supplementary Fig. 3. Variation in viremia kinetics between ZIKV strains after inoculation of a high virus dose. Time course of ZIKV viremia in AG129 mice inoculated on day 0 with 10^3 PFU of ZIKV. Viremia is expressed in log_{10}-transformed viral genome copies per ml of plasma (mean ± standard error). Each virus strain is represented by *n*=5 mice on each day, with the exception of day 6 for the Senegal_2011 and Senegal_2015 strains that are represented by *n*=10 mice. Source data are provided as a Source Data file.

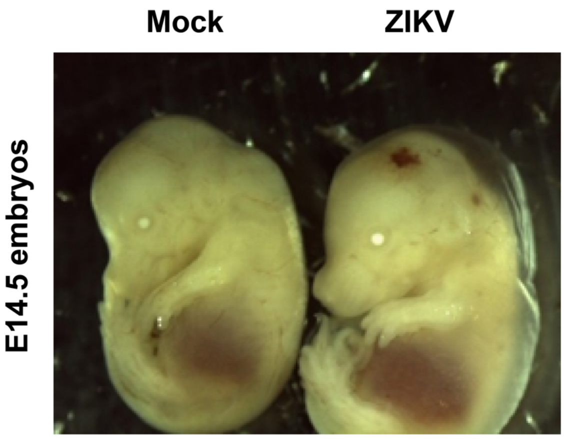

Supplementary Fig. 4. Intraplacental injections of ZIKV induce subcutaneous edema. Photos show representative E14.5 embryos after intraplacental injection of mock inoculum (left; *n*=10 embryos) or Senegal_2015 ZIKV strain (right; *n*=9 embryos) at E10.5.

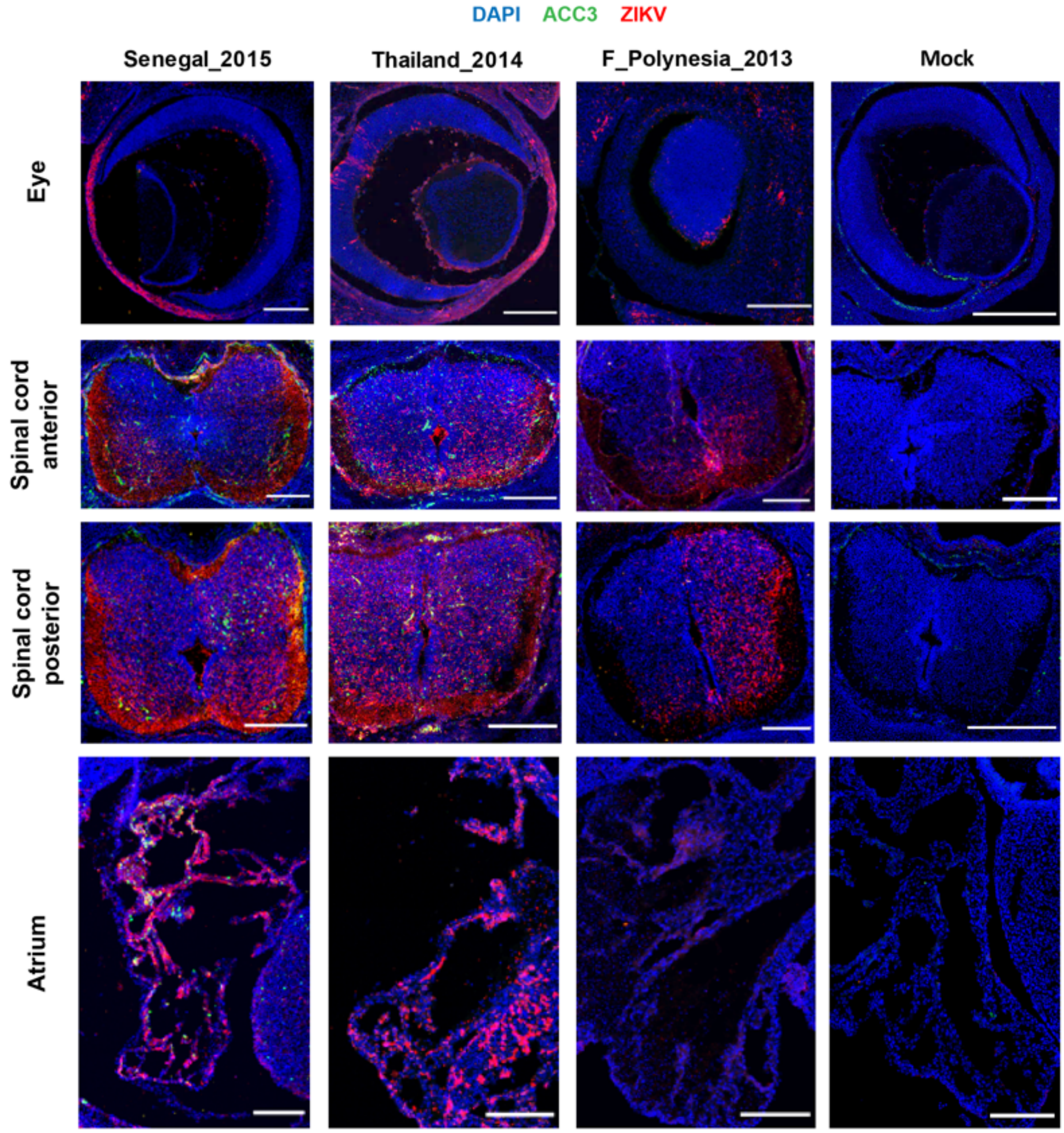

Supplementary Fig. 5. African and Asian ZIKV strains show comparable organs tropism. Immunocompetent mouse embryos were infected at E10.5 by intraplacental injection of 500-1,000 PFU of ZIKV and examined at E14.5 by microdissection. Immunolabeling of embryonic eye, anterior and posterior spinal cord and atrium sections representative of each ZIKV strain tested (*n*=3 embryos per virus strain). Blue, green and red colors indicate DAPI, anti-cleaved caspase 3 (ACC3) and ZIKV stainings, respectively. The scale bars represent 200 μm.
