# Supplementary material for: Recent African strains of Zika virus display higher transmissibility and fetal pathogenicity than Asian strains
Source: Nat Commun. 2021 Feb 10;12:916. doi: 10.1038/s41467-021-21199-z (PMC7876148; doi:10.1038/s41467-021-21199-z)
Supplement: Supplementary file 3 — Description of Additional Supplementary Files [file 41467_2021_21199_MOESM3_ESM.pdf]

Description of Additional Supplementary Files

File Name: Supplementary Software 1
Description: Custom code used to perform epidemiological simulations. The file contains the R script and complete instructions for installing and running the script.
