# Supplementary material for: Recent African strains of Zika virus display higher transmissibility and fetal pathogenicity than Asian strains
Source: Nat Commun. 2021 Feb 10;12:916. doi: 10.1038/s41467-021-21199-z (PMC7876148; doi:10.1038/s41467-021-21199-z)
Supplement: Supplementary file 5 — Reporting Summary [file 41467_2021_21199_MOESM5_ESM.pdf]

nature research

Corresponding author(s): Louis Lambrechts

Last updated by author(s): Jan 3, 2021

Reporting Summary

Nature Research wishes to improve the reproducibility of the work that we publish. This form provides structure for consistency and transparency in reporting. For further information on Nature Research policies, see our Editorial Policies and the Editorial Policy Checklist.

Statistics

For all statistical analyses, confirm that the following items are present in the figure legend, table legend, main text, or Methods section.

n/a	Confirmed	
☐	☒	The exact sample size (*n*) for each experimental group/condition, given as a discrete number and unit of measurement
☐	☒	A statement on whether measurements were taken from distinct samples or whether the same sample was measured repeatedly
☐	☒	The statistical test(s) used AND whether they are one- or two-sided<br>*Only common tests should be described solely by name; describe more complex techniques in the Methods section.*
☐	☒	A description of all covariates tested
☐	☒	A description of any assumptions or corrections, such as tests of normality and adjustment for multiple comparisons
☐	☒	A full description of the statistical parameters including central tendency (e.g. means) or other basic estimates (e.g. regression coefficient) AND variation (e.g. standard deviation) or associated estimates of uncertainty (e.g. confidence intervals)
☐	☒	For null hypothesis testing, the test statistic (e.g. *F*, *t*, *r*) with confidence intervals, effect sizes, degrees of freedom and *P* value noted<br>*Give P values as exact values whenever suitable.*
☒	☐	For Bayesian analysis, information on the choice of priors and Markov chain Monte Carlo settings
☒	☐	For hierarchical and complex designs, identification of the appropriate level for tests and full reporting of outcomes
☒	☐	Estimates of effect sizes (e.g. Cohen's *d*, Pearson's *r*), indicating how they were calculated

Our web collection on statistics for biologists contains articles on many of the points above.

Software and code

Policy information about availability of computer code

Data collection	Immunofluorescence images acquired by confocal microscopy were processed in ImageJ 1.42q 276, Fiji v2.0.0-rc-54/1.51 h and Zen (Blue edition, Carl Zeiss Microscopy GmbH) softwares.
Data analysis	Sequence data were analyzed in Trimmomatic v0.36, Bowtie2 v2.3.4.3, Ray v2.3.1-mpi, metaSPAdes, BLAST v2.2.40, MAFFT, ModelFinder, IQ-TREE v1.6.3 and FigTree v1.4.4. Statistical analyses were performed in JMP v10.0.2, GraphPad Prism v8.02 and R v3.6.0. Simulations used the custom code provided in Supplementary File 1. The R package nosoi is available from https://github.com/slequime/nosoi.

For manuscripts utilizing custom algorithms or software that are central to the research but not yet described in published literature, software must be made available to editors and reviewers. We strongly encourage code deposition in a community repository (e.g. GitHub). See the Nature Research guidelines for submitting code & software for further information.

Data

Policy information about availability of data

All manuscripts must include a data availability statement. This statement should provide the following information, where applicable:

- Accession codes, unique identifiers, or web links for publicly available datasets
- A list of figures that have associated raw data
- A description of any restrictions on data availability

Source data are provided as a source data file containing all the raw experimental data presented in the figures. Raw sequence data generated in this study were deposited in the European Nucleotide Archive database under accession number PRJEB39677 (https://www.ebi.ac.uk/ena/browser/view/PRJEB39677). Phylogenetic analysis of virus genomes used GenBank accession numbers MK241416, MF574587, KX198135, KU647676, KY693679, KU497555, MF794971, MK829154, MH882540, KY014295, MH063262, KY631494, KY693677, MF434522, MF801378, KU758877, KU937936, KY693680, KU509998, KX806557, LC191864, KU963796, MF036115, LC219720, MN190155, KY241695, MH013290, MK238037, KX051562, EU545988, KX377336, MN025403, MF510857, KU963574, KF268948,

MK105975, and KY288905.

Field-specific reporting

Please select the one below that is the best fit for your research. If you are not sure, read the appropriate sections before making your selection.

[x] Life sciences [ ] Behavioural & social sciences [ ] Ecological, evolutionary & environmental sciences

For a reference copy of the document with all sections, see nature.com/documents/nr-reporting-summary-flat.pdf

Life sciences study design

All studies must disclose on these points even when the disclosure is negative.

Sample size	No a priori sample-size calculation was performed. The sample size was typically 5-10 mice per group and 20-40 mosquitoes per group, as is generally the case in similar studies. Sample size was adjusted to capture the typical biological and experimental variation seen for the examined variables (Aubry F. et al. Science 2020; Zmurko J. et al. PLoS Negl. Trop. Dis. 2016; Gladwyn-Ng I. et al. Nat. Neurosci. 2018).
Data exclusions	Three extreme outliers were excluded from the mouse viremia data shown in Fig. 4c.
Replication	Robustness of experimental findings was supported by obtaining consistent results across multiple experiments, sometimes with different virus strains and/or doses. Experiments were generally repeated 2-3 times.
Randomization	Mice and mosquitoes were randomly allocated to experimental groups.
Blinding	Blinding was often impractical because the differences between mouse groups were visually obvious. When differences were not visually obvious, blinding was unnecessary because the biological measurements (viremia, viral load, weight) are objective and thus unlikely to be influenced by the experimenter.

Reporting for specific materials, systems and methods

We require information from authors about some types of materials, experimental systems and methods used in many studies. Here, indicate whether each material, system or method listed is relevant to your study. If you are not sure if a list item applies to your research, read the appropriate section before selecting a response.

Materials & experimental systems

n/a	Involved in the study	
[ ]	[x]	Antibodies
[ ]	[x]	Eukaryotic cell lines
[x]	[ ]	Palaeontology and archaeology
[ ]	[x]	Animals and other organisms
[ ]	[x]	Human research participants
[x]	[ ]	Clinical data
[x]	[ ]	Dual use research of concern

Methods

n/a	Involved in the study	
[x]	[ ]	ChIP-seq
[x]	[ ]	Flow cytometry
[x]	[ ]	MRI-based neuroimaging

Antibodies

Antibodies used	The primary antibodies were rabbit anti-cleaved caspase 3 (#9661, Cell Signaling Technologies), mouse anti-flavivirus group antigen (MAB10216, Merck Millipore), and goat anti-Iba1 (ab5076, Abcam). The secondary antibodies were donkey anti-rabbit conjugated with Alexa Fluor-488 (A-21206, Life Technologies), donkey anti-mouse conjugated with Alexa Fluor-555 (A-31570, Life Technologies), donkey anti-goat conjugated with Alexa Fluor-647 (A-21447, Life Technologies), and goat anti-mouse conjugated with Alexa Fluor-488 (A-11029, Life Technologies).
Validation	The primary antibodies were validated in several earlier studies (Fontaine A. et al. Sci. Rep. 2016; Baidaliuk A. et al. J. Virol. 2019; Gladwyn-Ng I. et al. Nat. Neurosci. 2018).

Eukaryotic cell lines

Policy information about cell lines

Cell line source(s)	The study used the Vero cell line (ATCC CCL-81), the Vero E6 cell line (ATCC CRL-1586) and the C6/36 cell line (ATCC CRL-1660).
Authentication	None of the cell lines used were formally authenticated.

Mycoplasma contamination	The cell lines were not tested for mycoplasma contamination.
Commonly misidentified lines (See ICLAC register)	None.

Animals and other organisms

Policy information about studies involving animals; ARRIVE guidelines recommended for reporting animal research

Laboratory animals	Mice were maintained under standard housing conditions (18-23°C, 14h:10h light:dark cycle and 40-60% relative humidity). AG129 mice (6- to 12-week-old, male) were obtained from Marshall BioResources (Hull, UK), 129S2/SvPas Ifnar1-knockout mice (10-week-old, male and female) were obtained from the Central Animal Facility of Institut Pasteur (Paris, France) and SWISS mice (6- to 12-week-old, female) were obtained from Janvier Labs (Saint Berthevin, France). Mosquitoes were maintained under controlled insectary conditions (28°±1°C, 12h:12h light:dark cycle and 70% relative humidity). Mosquito experiments used 2- to 7-day-old females from Aedes aegypti colonies originating from Barranquilla, Colombia (4th-5th generations), Saint François, Guadeloupe (9th generation) and La Lopé, Gabon (13th generation).
Wild animals	The study did not involve wild animals.
Field-collected samples	Mosquito colonies (Aedes aegypti) were originally established from wild specimens caught in Barranquilla, Colombia in 2017, Saint François, Guadeloupe in 2015 and La Lopé, Gabon in 2014. Mosquito colonies were maintained under controlled insectary conditions (28°±1°C, 12h:12h light:dark cycle and 70% relative humidity).
Ethics oversight	The study was approved by the French Ethical Committee Ile-de-France I, the Institut Pasteur Animal Ethics Committee, the French Ministry of Research, the French Ministry of Agriculture, and the Ethical Committees of the Animal Research Center of KU Leuven and University of Liège.

Note that full information on the approval of the study protocol must also be provided in the manuscript.

Human research participants

Policy information about studies involving human research participants

Population characteristics	Fresh human blood was obtained from healthy adult volunteers (seronegative for Zika virus) through the ICAReB biobanking platform of Institut Pasteur.
Recruitment	The experimenters of the study were blind to the recruitment of human subjects, which did not rely on any selection bias besides a negative Zika virus serology. All human subjects provided written informed consent.
Ethics oversight	Blood samples were supplied by the ICAReB biobanking platform within the CoSImmGen and Diagmicoll protocols, which were approved by the French Ethical Committee Ile-de-France I. The Diagmicoll protocol was declared to the French Research Ministry under reference 343 DC 2008-68 COL 1.

Note that full information on the approval of the study protocol must also be provided in the manuscript.
